# The “Id” Knows More than the “Ego” Admits: Neuropsychoanalytic and Primal Consciousness Perspectives on the Interface Between Affective and Cognitive Neuroscience

**DOI:** 10.3390/brainsci2020147

**Published:** 2012-04-17

**Authors:** Mark Solms, Jaak Panksepp

**Affiliations:** 1 Department of Psychology, University of Cape Town, Cape Town 7701, South Africa; 2 Department of VCAPP, College of Veterinary Medicine, Washington State University, Pullman, WA 99164, USA; E-Mail: jpanksepp@vetmed.wsu.edu

**Keywords:** affective consciousness, cognitive consciousness, brain evolution, mind evolution, emotions, perception, neuropsychoanalysis

## Abstract

It is commonly believed that consciousness is a higher brain function. Here we consider the likelihood, based on abundant neuroevolutionary data that lower brain affective phenomenal experiences provide the “energy” for the developmental construction of higher forms of cognitive consciousness. This view is concordant with many of the theoretical formulations of Sigmund Freud. In this reconceptualization, all of consciousness may be dependent on the original evolution of affective phenomenal experiences that coded survival values. These subcortical energies provided a foundation that could be used for the epigenetic construction of perceptual and other higher forms of consciousness. From this perspective, perceptual experiences were initially affective at the primary-process brainstem level, but capable of being elaborated by secondary learning and memory processes into tertiary-cognitive forms of consciousness. Within this view, although all individual neural activities are unconscious, perhaps along with secondary-process learning and memory mechanisms, the primal sub-neocortical networks of emotions and other primal affects may have served as the sentient scaffolding for the construction of resolved perceptual and higher mental activities within the neocortex. The data supporting this neuro-psycho-evolutionary vision of the emergence of mind is discussed in relation to classical psychoanalytical models.

## 1. Introduction

Our aim here is to introduce a novel way of integrating affective and cognitive aspects of conscious and unconscious brain processes, using a neuropsychoanalytic framework. Our starting point is the commonplace observation that different fields of inquiry use the terms “conscious” and “unconscious” in different ways, which prevents coherent discourse among the disciplines. The field at large has no standard definitions of these terms and their cognates. For instance, what is conscious “awareness”? Does the conscious subject need to have more than simple phenomenal experiences to have conscious “awareness”? Does “awareness” always imply a capacity for reflexive recognition of the fact that one is having experiences? Can we have (and empirically study) phenomenal consciousness without higher forms of consciousness that can report about awareness? We believe the answer to the last question has to be “yes”; otherwise we exclude all other animals from the circle of consciousness, which makes no evolutionary sense, especially given the abundant evidence for “rewarding” and “punishing” brain functions concentrated in various subcortical brainstem regions. 

To avoid such ambiguities, we start from the premise that the essential nature of consciousness is a foundational form of phenomenal experience, which in our view includes various affective states that can be monitored in animals by the rewarding and punishing properties of artificial evocation of such states with deep brain stimulation. From our perspective, the capacity to be aware of the environment and that one is the subject of such externally triggered experiences is already a higher cognitive function, which is ultimately mediated by the ability to reflect upon one’s subjective experiences. This hierarchical parsing enables one to be conscious in different ways—e.g., to feel happy and sad, without necessarily having the mental capacity to recognize that one is happy or sad, let alone to reflect upon the objective relations that caused this happiness or sadness. Being phenomenally conscious does not, by itself, require much cognitive sophistication at all. 

## 2. Synopsis of Our Overall Framework

Such modest conceptual distinctions permit the construction of evolutionarily sound scientific approaches to consciousness, which enable us, for example, to distinguish the brain structures supporting its phylogenetically ancient forms—which, based on abundant empirical studies, are affectively shared by all mammals—from those supporting the higher forms of consciousness that require reflexive and declarative abilities, which can only be studied systematically in creatures that speak for themselves. Failure to make such distinctions produces types of discourse that prematurely render lower brain processes “unconscious” simply because the forms of internally generated experiences are not often recognized, and easily spoken about, by higher, specifically-human forms of linguistically-based awareness. For example, certain mammalian species may have a great abundance of affectively and perceptually conscious phenomenal experiences, with very little capacity to reflect on those experiences. An evolutionarily tiered approach to consciousness also allows us to understand how it happens that strong affective states can occur without the subject of those states cognitively recognizing (“accepting”) the associated feelings. Moreover, since higher functions can inhibit lower functions in the brain (and *vice-versa*), we can see how certain forms of phenomenal experience may be temporarily rendered “unconscious” through active inhibitory influences. Strong emotions can also interfere with and disrupt cognitive processing; so it readily happens that individuals may experience strong emotional turmoil without having any subsequent insight into, or even memory of, those experiences. These examples could easily be multiplied.

In short, the complexity of our capacity to consciously and unconsciously process fluctuating brain states and environmentally linked behavioral processes requires some kind of multi-tiered analysis, such as Endel Tulving’s well-known parsing of consciousness into three forms: *anoetic* (unthinking forms of experience, which may be affectively intense without being “known”, and could be the birthright of all mammals), *noetic* (thinking forms of consciousness, linked to exteroceptive perception and cognition) and *autonoetic* (abstracted forms of perceptions and cognitions, which allow conscious “awareness” and reflection upon experience in the “mind’s eye” through episodic memories and fantasies) [1]. 

**Figure 1:**
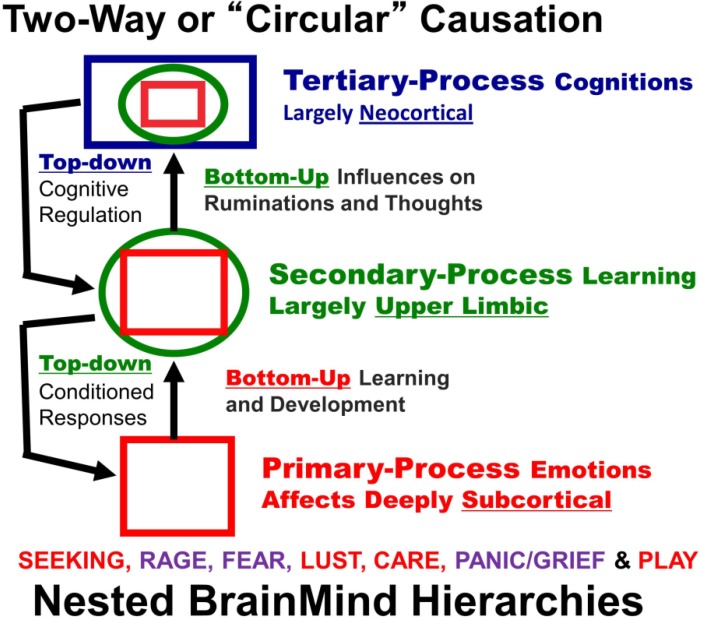
A schematic showing nested hierarchies of brain functions in which primary processes (red squares) influence secondary (green circles) and tertiary (blue rectangles) processes, which in turn exert top-down regulatory control. The seven primary process emotions are noted: positively valenced emotions highlighted in red (SEEKING, LUST, CARE and PLAY), and negative ones in purple (RAGE, FEAR and PANIC/GRIEF) [2,4].

This kind of a conceptual scheme can be readily overlaid on some major evolutionary passages of the brain, which roughly correspond to the evolution of (a) upper brainstem (up to the septal area), which permits *anoetic* phenomenal experiences, (b) lower subcortical ganglia and upper limbic structures (e.g., the cortical midline), which permit learning and *noetic* consciousness, and (c) higher neocortical functions (including all association cortices), which provide the critical substrates for the *autonoetic*, reflexive experiential blends that yield the stream of everyday awareness. Such multi-tiered parsing of consciousness (see Figure 1, adapted from [2]) enables us to recognize not only deeply unconscious brain processes, critical for behavior, but also brain processes that may be experienced at one level but not at another. This permits us to include in our scientific discourse the massive and ever-mounting evidence for the existence of various perceptual and affective forms of phenomenal consciousness in other animals [3,4]. This may enable us to develop better neuroevolutionary paradigms to scientifically unravel the fundamental biological nature of consciousness, as well as the differential natures of its many strange manifestations across multiple levels. 

Here we will address the multi-tiered complexities of consciousness from both modern cognitive and affective neuroscience perspectives. However, to properly contextualize these perspectives historically, we will frame selected aspects of the cognitive and affective data within a psychoanalytic frame of reference, which remains surprisingly relevant for the controversies under discussion here. Although the interregnum of “never-mind” behaviorism, which simply ignored the Black Box of the brain, interrupts the historical continuities, we hope that embedding our discussion in a clear account of seminal foundational issues that were explicitly considered at the outset of the modern neuroscientific era, will help us better understand what the splintered subfields of current neuroscience can and must still achieve. Our hope is that this will eventually yield a unified vision of how the mind actually works. 

To set the stage in a historical perspective, we contextualize our argument within the theoretical framework of classic psychoanalytic thought that may help highlight some provocative conceptual relationships to our approach. A close reading of Freudian thought, suggests he took a very similar approach to the mind. Even though Freud, a bench neuroscientist at the beginning of his career, did not have the wealth of neuroscientific knowledge we presently have, our objective analysis of the mental apparatus has distinct resemblances to his efforts, which emerged strictly from the subjective perspective. We offer this as an example of convergence, which hopefully promotes more careful empirical analyses of the affective foundations of the human mind than have been evident in so much of modern neuroscience, which typically offers a ruthlessly reductionistic view of the brain, as if an analysis of the mental apparatus is irrelevant for understanding what the brain does. 

## 3. Freud’s Notion of Unconscious Mental Processes

The notion that the brain knows more than it consciously admits can be traced back historically to the clinical and conceptual work of Sigmund Freud. He based this notion, which was considered radical at the time, upon observations he made of post-hypnotic suggestion and clinical states of dissociation, where behaviors were demonstrably caused by motivations of which the subject was not explicitly aware, some of which could subsequently be brought to awareness during psychological treatment. Since a scientific account of mental life cannot exclude unconscious cognitions with such demonstrable causal effects, Freud concluded that the objects of psychology must include unconscious processes, notwithstanding the conventional equation of “mind” with consciousness [5]. Thus, Freud devoted the remainder of his scientific life to studying the unconscious mental processes.

His first major conclusion was that such processes admitted of further differentiation. Some unconscious processes could be rendered conscious at will; others could not. Freud termed these processes “preconscious” and “unconscious” respectively. He then subdivided the unconscious processes into those that occurred outside of conscious awareness by dint of automatization (e.g., habituated skills), and those that were actively excluded from awareness by motivated resistances (e.g., electing not to think about something in order to avoid negative affective arousal). He termed these processes “descriptively” and “dynamically” unconscious respectively. The dynamically unconscious processes included some that were “repressed” from consciousness and some that never attained consciousness in the first place.

Freud’s second major conclusion was that these different grades of unconscious process displayed different functional properties. Dynamically unconscious processes were less constrained by realistic life considerations than preconscious or descriptively unconscious ones. The latter processes were, conversely, less influenced by affective states than the dynamically unconscious ones. On this basis Freud differentiated mental processes which obeyed what he termed the “reality principle” from those that obeyed the “pleasure principle,” with the latter being relatively unconstrained by inhibitory controls. In modern terms, they may be conceptualized as being activated by “free energy” [6,7]. Since the uninhibited processes were assumed to be phylogenetically older, and predominated in the juvenile mind, Freud termed them “primary”; the inhibited, realistic processes were described as “secondary” (which in Figure 1 are further distinguished into secondary, namely learned, and tertiary, namely thought-related, processes). 

Borrowing from Helmholtzian thermodynamics [8], Freud speculated that secondary processes [9] entail some form of “binding” of “free” energies, arising from the vital needs of the organism, although he admitted his ignorance of the underlying physiology of these processes. He inferred that the compulsive “free” energies press for immediate motor discharge (since they are unconstrained by realistic considerations) whereas “bound” energies, which are utilized in executive cognitive processes, give rise to more expedient, delayed responses. Freud imagined that such hypothetical energies “spread over the memory-traces of ideas somewhat as an electric charge is spread over the surface of a body” ([10], p. 60). Mental processes were therefore fundamentally conceptualized by Freud as being composed of (a) representations, activated by (b) drive energies (which he sometimes also called “quotas of affect”). These two mental elements were considered to be unconscious in themselves, and only to give rise to the phenomena of consciousness under certain conditions. 

We will develop the theme here that these two elements remain the foundational concepts of modern cognitive and affective science, but we will argue that the so-called “drive energies” that activate cognition are intrinsically conscious—although the transformations they are subject to frequently render them inaccessible to reflexive awareness. 

Freud formulated the conditional foundations of consciousness within a series of models of the functional architecture of the mind. In his first model [11] he attributed (unconscious) representational processes to a system of forebrain neurons that were distinguished from other neurons by their capacity for memory. He called this representational system “ψ”. Freud described this system as a “sympathetic ganglion” because its biological purpose was to associatively link endogenous needs (expressed as drives) with the external objects that satisfied them. The ψ system of neurons was split into “pallium” and “nuclear” divisions. The pallium division received its inputs via subcortical thalamic and cranial-nerve pathways from the sensory periphery (Freud called these pathways the “φ” system of neurons). The nuclear division of ψ received its inputs from the interior of the body, which Freud described as the “wellspring” of the mental apparatus, for the reason that it was constantly activated (or “cathected,” to use his term) by drive energies emanating from the relentless vital (survival and sexual) needs of the body. Tonic inhibition of this interoceptive division of ψ was accordingly assumed to be the physiological basis of executive control (the “ego”). 

Consciousness, which was attributed to a separate neuronal system (“ω”), was located at the motor end of the apparatus. The distinctive function of the ω system was to monitor the accumulation of drive energies within ψ. Increased drive tension generated feelings of unpleasure in ω; motor discharge, by contrast, generated pleasure. This affective-homeostatic function was, according to Freud, the primary purpose of consciousness. He therefore always insisted that affects were conscious by definition (see Section 11 below). Affect was the *raison d’être* of consciousness. However, consciousness was also extended to external sensory perception, by complicated mechanisms that revolved around the function of attention, which increased the level of activation of the exteroceptive division of ψ [12,13]. Consciousness accordingly occurred in two forms: (a) the form attached to interoceptive affects, and (b) the form attached to exteroceptive perceptions. Regarding the qualitative differences in the forms of consciousness, Freud stated only that the various modalities appeared to arise from different rhythms or patterns or “periods” (as opposed to degrees) of neuronal activity—which at that time was an inferred process, since organized neuronal activity had not yet been measured.

**Figure 2:**
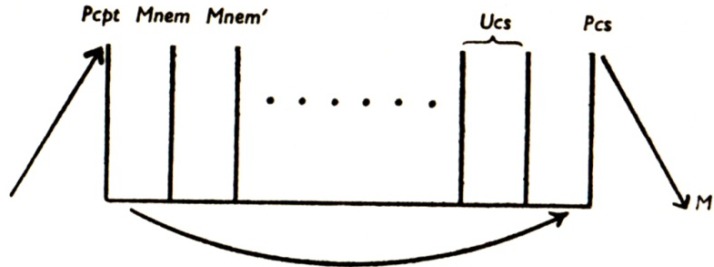
Freud’s second model of the mental apparatus. Pcpt (previously φ) = Perceptual system; Mnem (previously ψ) = Mnemic systems; Ucs = Unconscious system; Pcs = Preconscious system; M = Motor system [also known as Consciousness system, abbreviated Cs (previously ω)]. Reproduced from [5] with permission.

Freud’s second model of the mental apparatus [14] was essentially the same as his first, apart from the fact that the hypothetical neuronal systems were now given purely functional designations. This acknowledged Freud’s ignorance of the anatomy and physiology of the arrangements he had described, inferred as they were from psychological and behavioral rather than neuroscientific observations. Accordingly, the φ neurons became the “perceptual system” of the mind (abbreviated “Pcpt.”); the ψ neurons became “mnemic systems,” split into uninhibited and inhibited divisions, which thereby became the systems “unconscious” (abbreviated “Ucs.”) and “preconscious” (abbreviated “Pcs.”) respectively (see Figure 2 [15]); while the ω neurons became the system “consciousness” (abbreviated “Cs.”), still located at the motor end of the apparatus.

A significant revision was introduced in 1917 when Freud [16] combined φ and ω (the perceptual and motor systems adjacent to ψ) into a single integrated system for perceptual consciousness (“Pcpt.-Cs.”), on the grounds that all varieties of consciousness were at bottom perceptual. What distinguished the systems Pcpt. and Cs. was merely the sources of their stimuli and the modalities of perception they gave rise to. The classical sensory modalities, which registered the state of the outside world, were perceived on the “external surface” of the system Pcpt.-Cs., while affects, which registered the state of the apparatus itself, were perceived on the “internal surface” of this same, integrated system.

**Figure 3:**
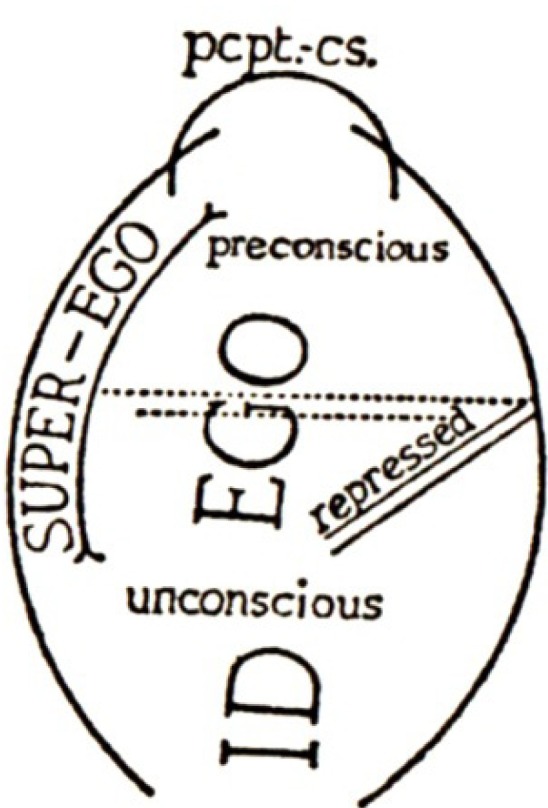
Freud’s final model of the mental apparatus. Pcpt.-Cs. = Perceptual-Consciousness system. Reproduced from [17] with permission.

This revision was retained in Freud’s final model [17]. The major purpose of his last revision was to recognize the fact that executive control did not coincide with any grade of consciousness (or preconsciousness). For example automatized “descriptively” unconscious processes, which never became conscious, were under inhibitory control (they were secondary processes; *cf.* [5,15]) and served the functional purposes of the reality principle. Likewise, the process of repression, despite being “dynamically” unconscious, served inhibitory purposes. Freud thus grouped all the inhibited grades of both conscious and unconscious processes under a single executive system—the “ego”—the distinguishing feature of which was its utilization of “bound” energies (which underpinned all “cognitive” processes); and he likewise grouped all the uninhibited (“instinctual” affective) processes under a single system utilizing “free” drive energy—the “id” (see Figure 3). 

By the end of Freud’s scientific life, therefore, the notion that “the brain knows more than it admits” revolved around the theory that bottom-up interoceptive pressures on the mind produced a set of primitive, compulsive drives (in the “id”) which aimed at immediate instinctual satisfaction, on the basis of affective-homeostatic imperatives regardless of the variable dictates of reality. Access by these influences to the executive motor system therefore had to be constrained, through top-down exteroceptive (“ego”) influences, perception and learning.

These theoretical developments, which introduced the very idea of unconscious mental processes to psychology, took place between 1894 and 1923. It is important to note that, as Freud shifted from a neurological to a functional description, his successive models of the mind still remained tethered to the body at three cardinal points: (a) the sense organs, (b) the vital needs, and (c) the motor system. Now, a century later, due to dramatic advances in the neurosciences, we are able to translate Freud’s functional descriptions of these bodily origins of the mind back into the language of anatomy and physiology. We do so in Section 5 below, in the spirit of Eric Kandel’s remark to the effect that Freud’s models still provide “the most coherent and intellectually satisfying view of the mind” that we have ([18], p. 505).

## 4. A Related Modern Conversation: There Are Two Bodily Origins of the Mind, and They Are Represented Differently

At the 12th International Neuropsychoanalysis Congress, held in Berlin in June 2011, on the topic of “Minding the Body”, Bud Craig, Antonio Damasio, Jaak Panksepp, Vittorio Gallese and Manos Tsakiris, among others, summarized the current state of knowledge as to how human mental functioning is embodied. In his closing remarks to the congress, Mark Solms pointed out that the speakers had referred to two different aspects of the body, without always distinguishing them. This can lead to confusion.

The first aspect of the body pivots around somatotopic maps on the cortical surface that are derived from sensory receptors on the surface of the body. This aspect of body representation corresponds most directly to the cortical homunculus. However, it does not coincide with somatosensory cortex alone. It also includes the projection zones of the other sensory modalities, which consist of topological maps of different sensory organs. It includes also the subcortical modality-specific thalamic and cranial nerve structures that link these terminal sense organs with the cortex. 

**Figure 4:**
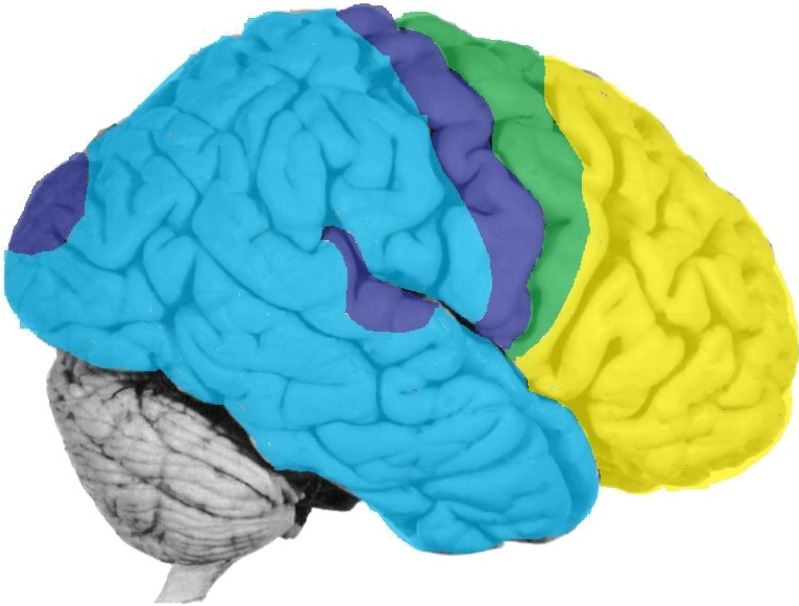
Dark blue = exteroceptive projection cortex; light blue = perceptual association cortex; green = motor projection cortex; yellow = motor association (executive) cortex.

The “body image” arises not in but rather from these modality-specific cortical maps. This aspect of bodily representation should therefore be equated also with the various perceptual streams arising from the projection zones and converging in association cortex. In this paper, we will call this aspect of bodily representation the “external body” for short (see Figure 4).

It is important to note that the brain mechanisms which represent the external body also represent other external objects. The external body is an object. It is the aspect of the body that one perceives when one looks outwards, at a mirror, for example. (“That thing is me”; it is “my body”.)

It should be remembered that motor maps, too, contribute to the external body image. The sense of possessing a three-dimensional body is determined not only by heteromodal sensory convergence but also by movement. Movement produces kinesthetic sensations, and may have intrinsic brain feelings of its own. The close relationship between movement and (muscle and joint) sensation is reflected in the anatomical proximity of the respective cortical zones: the somatosensory and motor projection areas form an integrated functional unit.

**Figure 5:**
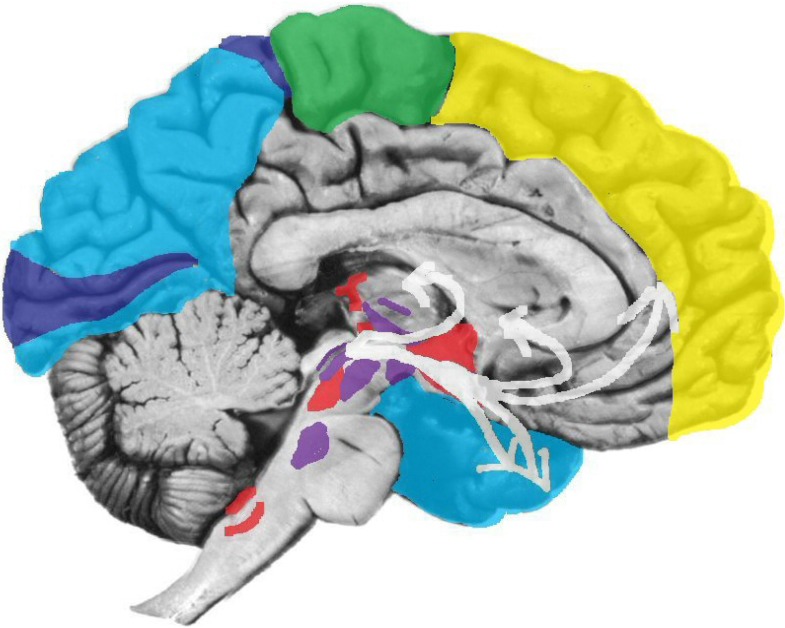
Red = some interoceptive nuclei; magenta = some ERTAS (arousal) nuclei; white = some basic emotion circuits.

The second aspect of the body is its internal milieu, the autonomic body, which is represented deeper and lower in the brain. The brain structures that represent this aspect of the body pivot around the hypothalamus but they also include the circumventricular organs, parabrachial nucleus, area postrema, solitary nucleus, and the like (see [19,20,21] for reviews). Analogous to what we said above about the motor cortex in relation to exteroception, these interoceptive structures, too, not only monitor but also regulate the state of the body (*cf.* homeostasis). Such subcortical network arousals may have phenomenal affective feelings of their own. In the present paper we will call this aspect of bodily representation the “internal body” for short (see Figure 5). 

Even at the level of the brainstem, the neural structures for the internal body are covered by those for the external body, just as the musculoskeletal body itself envelops the viscera. This largely reflects the fact that the evolution of coherent cerebral visceration is more ancient and foundational than ambulant movement for the somatic action apparatus. There is enormous evidence that primary-process emotional action coherence, and the raw feelings that concurrently emerge, arise from these subcortical circuits [4].

The brain mechanisms of the internal body function largely automatically, but they also arouse the external body to serve its vital needs in the external world. This is achieved through a network of upper brainstem and basal forebrain “arousal” structures known conventionally, but somewhat misleadingly, as the extended reticulo-thalamic activating system (ERTAS). This arousal system is constituted of many distinct long-axoned neuronal subsystems that include acetylcholine, epinephrine/norepinephrine, dopamine and serotonin systems, as well as a variety of neuropeptides [4,22]. In addition there is a complex internal structure to cerebral visceration processes (for overview see [23,24,25]). The primal roots of emotionality are grounded in these autonomic substrates.

It is important to note that an interdependent, hierarchical relationship therefore exists between the two aspects of the body. Considering the evolutionarily ancient roots of visceration, situated as they are more caudally and medially in the brain, there are reasons to believe that cerebral visceration (and hence emotionality) provided a bodily-coherence generating substrate for future brain developments, including the more cognitive domains. However, as some forward-looking scholars have long emphasized, from David Hume to Antonio Damasio, the emotional components are still critically important for the way the cognitive overlay operates.

Furthermore, it is becoming ever more evident that the internal body generates a very different type of consciousness from the consciousness associated with exteroceptive cortex. The interoceptive brainstem, along with diverse emotional networks, generates internal “states” rather than external “objects” of consciousness (see [4,20] for reviews). In other words, the internal body is not represented as an object of perception. Rather it gives rise to a background state of “being”; this aspect of the body is the *subject* of perception. We may picture this type of consciousness as the neurodynamic page upon which, or from which, exteroceptive experiences are written in higher brain regions. (This is also what binds experiences together; perception happens to a unitary, embodied subject; *cf.* the binding problem.)

It is important to note that these “states” of the body-as-subject involve not only varying levels of consciousness (e.g., sleep/waking) but also varying qualities of consciousness. Interoceptive consciousness, too, is phenomenal; it “feels like” something. Above all, the phenomenal states of the body-as-subject are experienced affectively. Affects, rather than representing discrete external events, are experienced as positively and negatively valenced states. Their valence is determined by how changing internal conditions relate to the probability of survival and reproductive success. At this level of the brain, therefore, homeostasis is inseparable from consciousness. Whereas the classical sensory modalities represent discrete external (knowledge-generating and objective) *noetic* happenings, affective consciousness represents diffuse internal (automatically evaluative and subjective) *anoetic* reactions to those happenings. Affectivity is, in this respect, a unique experiential modality. But that is not all it is; affectivity is an intrinsic property of the brain which is expressed in the emotions, and emotions are, above all, distinct forms of somatic motor discharge coordinated with supportive patterns of autonomic change. However, these emotional expressions also “feel like” something, in diversely valenced ways. The empirical evidence for the feeling component are simply based on the highly replicable fact that wherever in the brain one can artificially evoke coherent emotional response patterns with deep brain stimulation, those shifting states uniformly are accompanied by “rewarding” and “punishing” states of mind [2,4]. 

The keynote of affective consciousness is provided by the pleasure-unpleasure series, the motor expression of which is approach-withdrawal behavior. Feelings of pleasure-unpleasure, delight-distress—and the associated compulsive behaviors—are readily generated by stimulating as low in the brainstem as the periaqueductal grey (PAG). The generating of such reactions is thought to be the biological “purpose” of consciousness [4,19]. By attributing valence to experience—determining whether something is “good” or “bad” for the subject, within a biological system of values—affective consciousness (and the behaviors it gives rise to) intrinsically promotes survival and reproductive success. This is what consciousness is for. It also motivates the cognitive controls that emerged during further encephalization. This provides increasingly sophisticated mental strategies for controlling behavior on the basis of what animals can do with external *noetic* information-processing.

To this end, arising from the PAG and ascending into the limbic forebrain, which reciprocally provides many descending controls, are various instinctual motivational circuits. These are also known as the circuits for “basic emotion”. There are several classifications of these emotions. The best-known examples are those that generate (a) appetitive foraging, (b) consummatory reward, (c) freezing and flight, (d) aggressive attack, (e) nurturant care, (f) separation distress, and (g) rough-and-tumble play (see [4] for detailed review). The circuits for these basic emotions have been given special names (see below). Further analysis of their trajectories, neurochemistries, and neurodynamics, provide clear targets for modern neuroscience tools that have the potential to reveal the constitution of affective phenomenal consciousness within the brain. Our concurrent working assumption is that this type of consciousness provided some critical raw materials for the construction of cognitive forms of experience, in higher regions of the brain. 

It is important to note that each of the instinctual circuits generates not only stereotyped behaviors but also diverse feeling states, such as expectant interest, orgasmic delight, trepidatious fear, destructive rage, loving affection, sorrowful grief, and exuberant joy. Again, the critical evidence for this comes from the fact that these emotional states can be aroused by artificial electrical and chemical stimulation of the specific structures along these circuits, and they serve as specific rewards and punishments in the control of simple approach and withdrawal behaviors. The circuits for these basic emotions are conserved across the mammalian series, and they admit of considerable chemical specificity. It is important to note that these instinctual motivations are genetically built into the cross-mammalian foundations of the brain [4,19]. They are no less innate than the vital evolutionary survival and sexual needs which gave rise to them. They are unconditioned “tools for living”. The emotional learning associated with these instincts is secondary to the instincts themselves (see Figure 1), permitting us to envision novel “Laws of Affect” that control learning.

To be clear: subcortical affective processes come in at least three major categorical forms; (a) the homeostatic internal bodily drives (such as hunger and thermoregulation); (b) the sensory affects, which help regulate those drives (such as the affective aspects of taste and feelings of coldness and warmth); and (c) the instinctual-emotional networks of the brain, which embody the action tools that ambulant organisms need to satisfy their affective drives in the outside world (such as searching for food and warmth). These instinctual “survival tools” include: foraging for resources (SEEKING), reproductive eroticism (LUST), protection of the body (FEAR and RAGE), maternal devotion (CARE), separation distress (PANIC/GRIEF), and vigorous positive engagement with conspecifics (PLAY). This evidence based affective neuroscience taxonomy [2,4] uses a capitalized nomenclature to distinguish the identified primary instinctual-affective subcortical networks of the brain from the various colloquial blends of everyday cognitive awareness, namely the tertiary processes of Figure 1. Our main goal in this paper is to provide a more solid foundation of the primary-process emotional mind for the clinical, cognitive and social neurosciences than currently exists.

## 5. Exteroceptive Ego; Interoceptive Id

**Figure 6:**
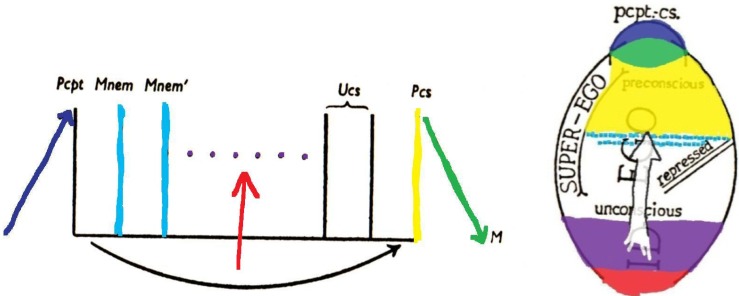
The preceding Figure 2 and Figure 3 are colored here to show some correspondences with the brain structures indicated by the same colors in Figure 4 and Figure 5.

Having reviewed the two ways in which the body is represented in the brain, it is easy to recognize in the data the two major mental systems that were described in the classical Freudian models reviewed before. The external body corresponds to the “ego”, the internal body is the “id” (see Figure 6).

Freud himself said as much. About the bodily origin of the “ego” Freud wrote this:

“The ego is first and foremost a bodily ego; it is not merely a surface entity, but is itself the projection of a surface. If we wish to find an anatomical analogy for it we can best identify it with the ‘cortical homunculus’ of the anatomists, which stands on its head in the cortex, sticks up its heels, faces backwards and, as we know, has its speech-area on the left-hand side”.

([17], p. 26)

He elaborated:

“The ego is ultimately derived from bodily sensations, chiefly from those springing from the surface of the body. It may thus be regarded as a mental projection of the surface of the body, besides, as we have seen above (Figure 3), representing the superficies of the mental apparatus.”

About the bodily origin of the “id”, Freud wrote:

“The id, cut off from the external world, has a world of perception of its own. It detects with extraordinary acuteness certain changes in its interior, especially oscillations in the tension of its instinctual needs, and these changes become conscious as feelings in the pleasure-unpleasure series. It is hard to say, to be sure, by what means and with the help of what sensory terminal organs these perceptions come about. But it is an established fact that self-perceptions—coenesthetic feelings and feelings of pleasure-unpleasure—govern the passage of events in the id with despotic force. The id obeys the inexorable pleasure principle”.

([26], p. 198)

The word “instinctual” here is a mistranslation of Triebe. A Trieb is a “drive”. Freud clearly defined what he meant by the term:

“An ‘instinct’ [Trieb] appears to us as a concept on the frontier between the mental and the somatic, as the psychical representative of the stimuli originating from within the organism and reaching the mind, as a measure of the demand made upon the mind for work in consequence of its connection with the body”.

([27], pp. 121–122)

It is evident that Freud himself readily “localized” the different bodily derivations of the ego and the id. We have only added anatomical detail (in Section 3), and clarified that there are within-brain needs, all of which distinctly arouse the body, and when aroused, continue to be modulated by the body. It is easy to recognize the functional equivalence between the brain mechanisms for exteroceptive representation and the bodily ego, on the one hand, and between those for interoceptive drive and the id, on the other. This applies equally to the interdependent hierarchical relationship between the two systems. These concepts therefore apply also to the brain processes that mediate bodily homeostasis and to those for the basic emotions, about which Freud said less. Still, Freud did envision the foundations of basic emotion in ways that reflect modern views:

“And what is an affect in the dynamic sense? It is in any case something highly composite. An affect includes in the first place particular motor innervations or discharges and secondly certain feelings; the latter are of two kinds—perceptions of the motor actions that have occurred and the direct feelings of pleasure and unpleasure which, as we say, give the affect its keynote. But I do not think that with this enumeration we have arrived at the essence of an affect. We seem to see deeper in the case of some affects and to recognize that the core which holds the combination we have described together is the repetition of some particular significant experience. This experience could only be a very early impression of a very general nature, placed in the prehistory not of the individual but of the species”.

([28], p. 395)

## 6. The Exteroceptive Fallacy

The close parallelism between the brain mechanisms for the external and internal aspects of body representation, on the one hand, and the functional properties of the ego and id on the other, reveal a stark contradiction between current affective neuroscience concepts of mind and those of Freud. In this respect Freud inaugurated the conflation of unconscious processes with cognitive unawareness of instinctual consciousness, thereby prematurely relegating unmonitored affective processes into the “unconscious” category (see [29], and the accompanying commentaries). As we will see, this practice oversimplifies the varieties of conscious and unconscious processes that actually exist.

To begin to expose this problem, we need to point out that Freud never questioned the classical assumption that consciousness was a cortical function:

“What consciousness yields consists essentially of perceptions of excitations coming from the external world and of feelings of pleasure and unpleasure which can only arise from within the mental apparatus; it is therefore possible to assign to the system Pcpt.-Cs. a position in space. It must lie on the borderline between inside and outside; it must be turned towards the external world and must envelop the other psychical systems. It will be seen that there is nothing daringly new in these assumptions; we have merely adopted the views on localization held by cerebral anatomy, which locates the ‘seat’ of consciousness in the cerebral cortex—the outermost, enveloping layer of the central organ. Cerebral anatomy has no need to consider why, speaking anatomically, consciousness should be lodged on the surface of the brain instead of being safely housed somewhere in its inmost interior”.

([30], p. 24)

Freud recognized that consciousness also entailed an interoceptive, affective aspect. He even suggested that this aspect defined the original biological “purpose” of consciousness ([31], p. 220). That is why Antonio Damasio was moved to say that “Freud’s insights on the nature of affect are consonant with the most advanced contemporary neuroscience views” ([32], p. 38). 

But it is clear from the above quotation that even the affective aspect of consciousness was, for Freud, “lodged on the surface of the brain”. Here he states this view even more explicitly:

“The process of something becoming conscious is above all linked with the perceptions which our sense organs receive from the external world. From the topographical point of view, therefore, it is a phenomenon which takes place in the outermost cortex of the ego. It is true that we also receive information from the inside of the body—the feelings, which actually exercise a more peremptory influence on our mental life than external perceptions; moreover, in certain circumstances the sense organs themselves transmit feelings, sensations of pain, in addition to the perceptions specific to them. Since, however, these sensations (as we call them in contrast to conscious perceptions) also emanate from the terminal organs and since we regard all these as prolongations or offshoots of the cortical layer, we are still able to maintain the assertion made above. The only distinction would be that, as regards the terminal organs of sensation and feeling, the body itself would take the place of the external world”.

([26], pp. 161–162)

In making this assumption Freud followed a long tradition, which continues to this day. Consider for example the following remark made by Joseph LeDoux:

“When electrical stimuli applied to the amygdala of humans elicit feelings of fear (see Gloor, 1992), it is not because the amygdala ‘feels’ fear, but instead because the various networks that the amygdala activates ultimately provide working memory with inputs that are labeled as fear. This is all compatible with the Freudian notion that conscious emotion is the awareness of something that is basically unconscious”.

([33], p. 46)

The latest incarnation of this “corticocentric” tradition is the work of Bud Craig [34]. He believes there is a cortical projection zone for the internal body, in the posterior insula, which he describes as the basis of the body-as-subject, the “self” (precisely the function we have attributed above, on the basis of a different research tradition, to the upper brainstem). We present this critique from the perspective that the locus of subjectivity does not reside in the insula, while conceding that many sensory affects (e.g., disgust)—as opposed to primary-process basic emotions [2,4,35]—and probably various other bodily sensations, along with certain sensory affects, are indeed processed by the insula [35].

## 7. Consciousness without Cortex

Recent research demonstrates unequivocally that the corticocentric view of consciousness (and the subjective “self”) is mistaken. Consider the following interview, reported at our Berlin congress by Damasio [36], concerning a patient in whom the insula was bilaterally (totally) obliterated by herpes simplex encephalitis. According to Craig’s view [34], this patient should lack subjective, affective selfhood; he should lack the very page upon which experience is written. But this was not the case:

Q: “Do you have a sense of self?”

A: “Yes, I do.”

Q: “What if I told you that you weren’t here right now?”

A: “I’d say you’ve gone blind and deaf.”

Q: “Do you think that other people can control your thoughts?”

A: “No.”

Q: “And why do you think that’s not possible?”

A: “You control your own mind, hopefully.”

Q: “What if I were to tell you that your mind was the mind of somebody else?”

A: “When was the transplant, I mean, the brain transplant?”

Q: “What if I were to tell you that I know you better than you know yourself?”

A: “I would think you’re wrong.”

Q: “What if I were to tell you that you are aware that I’m aware?”

A: “I would say you’re right.”

Q: “You are aware that I am aware?”

A: “I am aware that you are aware that I am aware.”

**Figure 7:**
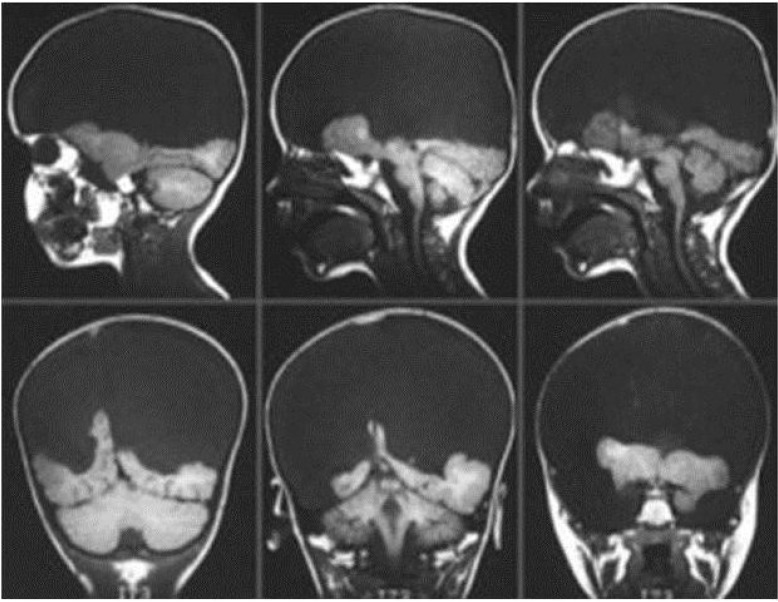
A typical hydranencephalic brain. (Reprinted with permission of the American College of Radiology [40]. No other representation of this material is authorized without expressed, written permission from the American College of Radiology).

**Figure 8:**
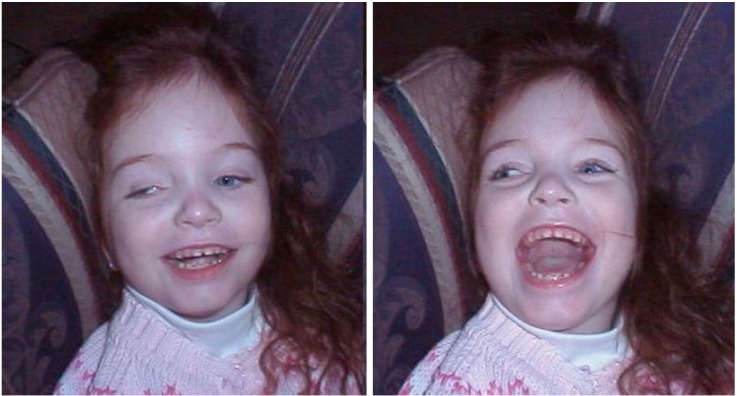
The emotional reaction of a young hydranencephalic girl. (We thank Bjorn Merker for the use of these photographs, reproduced with permission of the child’s mother [41]).

This case disproves only Craig’s restricted (insular) version of the corticocentric theory. What about the rest of the cortex? In preclinical animal models, the removal of the neocortex has long been known to spare emotionality. Indeed, not only are the rewarding effects of subcortical brain stimulations demonstrably preserved in decorticated creatures, these animals are actually more emotional than normal [37,38]. The most strikingly concordant human evidence to emerge in recent years, relevant to this broader question, concerns a condition called hydranencephaly, in which the cerebral cortex as a whole is destroyed *in utero* (usually due to infarction in the entire territory of the anterior cerebral circulation) [39]. Autopsy studies reveal that islands of cortex which may be preserved in such cases (see Figure 7) are functionally disconnected from the thalamus due to destruction of the linking white matter. The surviving cortical fragments are also gliotic, and therefore completely non-functional. This is confirmed by the clinical observation that, although visual cortex is preserved, the patients are blind [3]. However, the subcortical networks are functional; thus, the children are markedly emotionally functional human beings (see Figure 8).

“They express pleasure by smiling and laughter, and aversion by ‘fussing’ arching of the back and crying (in many gradations), their faces being animated by these emotional states. A familiar adult can employ this responsiveness to build up play sequences predictably progressing from smiling, through giggling, to laughter and great excitement on the part of the child” ([3], p. 79).

They also show basic emotional learning. They:

“take behavioral initiatives within the severe limitations of their motor disabilities, in the form of instrumental behaviors such as making noise by kicking trinkets hanging in a special frame constructed for the purpose (‘little room’), or activating favorite toys by switches, presumably based upon associative learning of the connection between actions and their effects. Such behaviors are accompanied by situationally appropriate signs of pleasure and excitement on the part of the child.”.

[3]

Although there is in these children significant degradation of the types of consciousness that are normally associated with external perception, there can be no doubt that they are conscious, both quantitatively and qualitatively. They are not only awake and alert, but also experience and express a full range of instinctual emotions. The raw affective self is, in short, fully present. The gold standard for affects in animals is that learned “reward” and “punishment” effects can be evoked by stimulating brain areas that arouse intense emotional displays, as can be seen in such children, as well as in decorticated animals. The fact that cortex is essentially absent in these cases proves unequivocally that affective consciousness is both generated and felt subcortically. This contradicts the theoretical assumptions of LeDoux and Craig quoted above, and those of Freud. Affective consciousness is not dependent on working memory being provided with unconscious subcortical inputs that are only then “labeled” as emotions. It is an intrinsic function of lower regions of the brain.

And this does not apply only to affective consciousness.

## 8. All Consciousness Is Endogenous

The “state” of consciousness as a whole is generated in the upper brainstem. We have known this for many years. A mere decade after the death of Freud, Moruzzi and Magoun [42] first demonstrated that global consciousness, in the sense measured by EEG activation, is generated not by exteroceptive stimuli but endogenously, in a part of the upper brainstem then called the “reticular activating system”. This was quickly confirmed by Penfield and Jasper, who recognized in absence attacks (mentioned above) “a unique opportunity to study the neuronal substratum of consciousness” ([43], p. 480). Their extensive studies led them to the conclusion that obliteration of consciousness could only be reliably evoked by restricted damage to such upper brainstem sites (which they termed the “centrencephalic system”). They were also impressed by the fact that removal of large expanses of cortex under local anaesthetic, even total hemispherectomy, had limited effects on consciousness. Cortical removal did not interrupt the presence of the conscious “self”, of conscious being, it merely deprived the patient of “certain forms of information” ([43], p. 65). Lesions in the region of the upper brainstem, by contrast, totally and rapidly destroyed consciousness, just as the absence seizures did. These observations demonstrated a point of fundamental importance: all consciousness ultimately derives from upper brainstem sources. Contrary to LeDoux and the other corticocentric theorists: all the cortical varieties of consciousness depend upon the integrity of these subcortical structures, not the other way round. This in not to deny that higher cortical regions add much to consciousness. Of course they do. But the evolutionary “roots” of consciousness are to be found elsewhere, and they are probably affective [4,44].

The classic observations that underpin this important conclusion have stood the test of time, with greater anatomical precision being added (see [24] for review). Significantly, the PAG appears to be a nodal point in the “centrencephalic system”. This underscores the single fact that has changed in modern conceptions of this system: the brainstem structures that generate conscious “state” are not only responsible for the degree but also for the core quality of subjective being. The primal conscious “state” of mammals is intrinsically affective. It is this realization that will revolutionize consciousness studies in future years [4,21]. 

To put it bluntly: consciousness is generated in the id. The classical conception is turned on its head. The cortex was not the initial generator of consciousness, but this is not widely recognized in consciousness studies. As the late Paul Grobstein said: “Is ‘reflective awareness’ a ‘luxury’ on top of consciousness? Or might it be that without which there is no internal experience at all?” [45]. This is the classic problem in consciousness studies that still prevents many scholars from accepting the evidence based conclusion that “reflective awareness” is not the sine qua non of subjective experience. Failure to accept the evidence, still allows many to assume that we have no right to conclude that human infants and other animals that can’t speak have affective experiences. Of course, deep science does not indulge in concepts such as “proof” but only in the “weight of evidence”; with that as the cardinal rule for scientific reasoning, the evidence for affective experiences in all non-speaking mammals has been quite overwhelming for a while [2,4,44]. Perhaps this line of reasoning will soon need to be extended to some invertebrates [46]. In contrast, there is no evidence that living neocortex alone, without subcortical supports, can have any subjective experiences at all [47,48]. 

## 9. Mental Solids

What, then, does cortex contribute to consciousness? It is clear from the facts just reviewed that the consciousness attached to core affective states is not really intrinsic to the cortex but rather derives from deep subcortical sources. Neocortex without a brainstem can never be conscious. Although neocortex surely adds much to refined perceptual awareness, initial perceptual processing appears to be unconscious in itself (*cf.* blindsight) or it may have qualities that we do not readily recognize at the level of cognitive consciousness. Exteroceptive sensory systems at the subcortical (e.g., tectal) level may have little more than an affective and bodily-orientation feel to them. An appropriate formulation of the relationship between perception and consciousness (which is, as we now know, endogenous, subjective and fundamentally interoceptive in an affective kind of way) might therefore be: “I feel like this about that” (where “this” is consciousness and “that” is perception). Consciousness is, to use Damasio’s term, thereby *extended* onto exteroception. It is possible that perceptual and higher cognitive forms of consciousness emerged in the neocortex upon an evolutionary foundation of affective consciousness [39,49]. In other words, *anoetic* phenomenal experiences may have emerged before *noetic* forms of consciousness in brain-mind evolution [50].

Moreover, much of what we have traditionally thought to be unconditioned about exteroceptive consciousness is actually learned. This has been well demonstrated by the research of Mriganka Sur, which shows that total removal of “visual” cortex in fetal mice (*in utero*) does not impair their adult vision at all, and redirecting visual input from occipital cortex to auditory cortex in ferrets leads to reorganization of the latter tissue to support completely competent vision (for review, see [51]). Clearly, from a corticocentric viewpoint, this either means that sensory perception is completely learned, or that perceptual functionality is completely controlled by subcortical structures, with subtle developmental extensions of affective experience perhaps being the foremost vehicle. In short, one of the great mistakes of modern cognitive neuroscience may be the assumption that cortical consciousness is built on intrinsic “hard-wired” cognitive computational principles. The resolution of conscious experiences in the neocortex may be largely learned developmental/epigenetic functions of the brain. For instance, the critical originating features of supposedly intrinsic cognition capacities, like the so-called “language instinct”, are more likely to be deeply affective—perhaps based on social-emotional “urges to communicate” feelings [52].

The fundamental contribution of cortex to consciousness in this respect is stabilization (and refinement) of the objects of perception and generating thinking and ideas. This contribution derives from the unrivalled capacity of cortex for representational forms of memory (in all of its varieties, both short- and long-term). To put it metaphorically, cortex transforms the fleeting, fugitive, wave-like states of consciousness into mental solids. It generates objects. (Freud called them “object presentations”.) Such stable representations, once established, can be innervated both externally and internally, thereby generating objects not only for perception but also for cognition. To be clear: the computations and memories underlying these representational processes are unconscious in themselves; but when consciousness is extended to them, it (consciousness) is transformed by them into something stable, something that can be thought, something in the nature of crystal clear perceptions that are transformed into ideas in working memory.

When we say that conscious perception expresses the formula “I feel like this about that” we are knowingly invoking Freud’s idea that the forebrain is a “sympathetic ganglion”, in the sense that exteroceptive consciousness and learning reflect and serve interoceptive needs. Learning arises from associations between interoceptive drives and exteroceptive representations, guided by the feelings generated by the affective experiences aroused by those representations. This is why they become conscious; the embodied subject must evaluate them. (The associations are to a large extent determined by the unconditioned categories of basic affects, but the representations themselves are not.) 

The stability of such representations then enables them to be used to guide subsequent conscious behavior (it enables them to be “held in mind”). The prototype for this in Freud’s conception was “wishing” (e.g., Berridge [53] calls it “wanting”), which was in the first instance regulated by the “pleasure principle” but energized by a pervasive urge to seek resources, from nuts to knowledge, so to speak. Instinctual affective bodily drives and emotions are initially objectless (*cf.* the “SEEKING” concept of Panksepp [4,44]), but sympathetic associative learning rapidly leads to remembered objects of desire “coming to mind”. In other words, wished-for (or feared, *etc.*) objects are rendered conscious by dint of their “incentive salience” (determined by their biological meaning to the subject as in the way SEEKING leads to interaction with the pleasure-unpleasure aspects of the environment, which is the ultimate basis of consciousness). In this way, if left to its own devices, the pleasure principle would produce what Freud termed hallucinatory wish fulfillments (the prototype of “primary process” cognition). Hence, the evolutionary and developmental pressure to constrain incentive salience in perception through prediction-error coding (the “reality principle”—a higher, tertiary order brain-mind process, in our terminology (Figure 4)) places inhibitory constraints on action. Such error coding must, again, be regulated by the homeostatic (affective) function of consciousness, which determines the biological valence of perceptions. The resultant inhibition—which perforce occurs at the motor end of the apparatus, where delayed responses must be sequenced—requires tolerance of frustrated affects, but it secures more efficient drive satisfaction in the long run (Freud called this the “constancy principle”). This defines the essence of the executive function expressed in working memory, in the sense that we generally theorize it today (a sense that Freud would have called secondary process thinking, which he also described as “virtual action”).

Freud’s secondary process, as we know from Section 3, involved “binding” of “free” drive energies. This created a reservoir of tonic mental energies, utilized for functions (like thinking) that Freud attributed to the “ego”. Carhart-Harris and Friston [54] recently equated this reservoir with the default mode network. In fact, Friston’s work is grounded in the same Helmholtzian conception that Freud’s was. His model of the Bayesian brain (in terms of which prediction-error or “surprise”, equated with “free energy”) is minimized through the encoding of better models of the world leading to better predictions [6] is therefore, in principle, entirely consistent with the model outlined here. 

It is important to note that in this model, prediction error (mediated by the sensory affect of surprise), which increases incentive salience (and therefore conscious “presence” of the self) in perception, is a “bad” thing, biologically speaking. The more veridical the brain’s generative model of the world, the less surprise (the less salience, the less consciousness, the more automaticity), the better. Freud called this the “Nirvana principle”. We shall return to it in Section 10.

Before we can do so, however, we must point out that secondary process thinking entails an important additional feature which may also be attributed to cortex. The wished-for object presentations that literally “come to mind” in primary process (hallucinatory) thinking are, according to Freud, re-represented at a different level in bound, secondary process thinking (which from an evolutionary brain perspective, we call tertiary-process). He called this level of representation “word presentation”. The value of word presentations for Freud was that—although they, like all cognitive representations, are originally derived from perception (in this case, hearing)—their symbolic nature enables them to represent abstract relations between the concrete objects of thought (“which is what specially characterizes thoughts, and cannot be given visual expression” ([17], p. 21)). This renders secondary process thinking far more efficient than the primary process. It also renders it “declarative”.

This is the contribution of cortex. Indeed, as far as we know, all cortical functional specializations are developmental/epigenetic. The columns of cortex are initially almost identical in neuronal architecture, and the famous differences in Brodmann’s areas probably arise from use-dependent plasticity. Metaphorically, cortical columns resemble the monotonous random-access memory (RAM) chips of digital computers. Can intrinsic biological consciousness originate there? Can the subjective aspects of mind really be computed? There is no consistent body of credible data to support either of these guiding assumptions of modern cognitive science. The prevailing Computational Theory of Mind seems fundamentally flawed. It remains a torso in search of a sophisticated neurobiological Affective Theory of Mind. The neocortex, the supposed repository of consciousness, is intrinsically unconscious, notwithstanding its remarkable capacity to generate the detailed and refined “mental solids” that obscure all else from view.

## 10. The Reflexive Ego

We said in Section 2 that external body representation is made of the same “stuff” as the representation of other objects. The external bodily “self” is represented as a thing—“my body”—and is inscribed on the page of consciousness (derived from the internal body-as-subject) in much the same way as other objects. It is, in short, an external, stabilized, detailed representation of the subject of consciousness. It is not the subject itself. It is important to recognize that this conception of the self is an illusion, albeit an everyday one. The external body is not the owner or locus of consciousness. It is not really the subjective self; it is an objective representation of the self.

The subject of consciousness identifies itself with this external bodily representation in much the same way as a child might project itself into the animated figures she controls in a computer game. The representations rapidly come to be treated as if they were the self, but in reality they are not.

Here is some experimental evidence for the counterintuitive relation between the self and its external body. Petkova and Ehrsson [55] reported a series of “body swap” experiments in which cameras mounted over the eyes of other people or mannequins, transmitting images from the their viewpoint to goggles mounted over the eyes of experimental subjects, rapidly created the illusion in the experimental subjects that the other body or mannequin was their own body. This illusion was so compelling that it persisted even when the subjects (projected into the other bodies) shook hands with their own bodies. The existence of this illusion was demonstrated objectively by the fact that when the other (illusory own) body and one’s own body were both threatened with a knife, the emotional reaction (measured by heart rate and galvanic skin response) was greater for the illusory body. 

The well-known “rubber hand illusion” demonstrates the same relation between the self and the external body, albeit less dramatically. So does the inverse “phantom limb” phenomenon. The anatomical basis of such phenomena (which place Freud’s theory of “narcissism” on a new empirical footing) may be equated with the well-known fMRI findings to the effect that the shape and size of somatosensory and motor cortical homunculi (the acknowledged locus of Freud’s “bodily ego”) can be readily manipulated, and even extended to include inanimate tools.

The secondary nature of external bodily representation is further demonstrated by many well-known “mirror neuron” phenomena. Gallese’s group has recently shown, for example, that schizophrenic patients are unable to reliably differentiate between their own movements and those of others, on the basis that mirror neuron activity (which generates cortical mirages of the own body moving when somebody else’s movements are observed), is not controlled by frontal inhibition in these patients [56].

The above phenomena demonstrate firstly that the external body is not a subject but an object, and secondly that it is perceived in the same register as other objects. Something has to be added to simple perception before one’s own body is differentiated from others.

In this connection, the role of prefrontal cortex in reflexive consciousness (a.k.a. secondary consciousness, access consciousness, declarative awareness, *etc.*) is surely germane. So is the role of prefrontal cortex in verbal re-representation (Figure 5 and Figure 6). This level of representation (a.k.a. higher-order thought) enables the subject of consciousness to separate itself as an object from other objects. We envisage the process involving three levels of experience: (a) the subjective or phenomenal level of the *anoetic* self as affect, a.k.a. first-person perspective; (b) the perceptual or representational level of the *noetic* self as an object, no different from other objects, a.k.a. second-person perspective; (c) the conceptual or re-representational level of the autonoetic self in relation to other objects, *i.e.*, perceived from an external perspective, a.k.a. third-person perspective. 

The self of everyday cognition is therefore largely an abstraction. That is why the self is so effortlessly able to think about itself in relation to objects, in such everyday situations as “I am currently experiencing my self looking at an object”.

The unrecognized gap between the primary subjective self and the re-representational abstracted self causes much confusion. Witness the famous example of Benjamin Libet recording a delay of up to 400 ms between the physiological appearance of premotor activation and the voluntary decision to move. This is typically interpreted to mean that free will is an illusion, when in fact it shows only that reflexive re-representation of the self initiating a movement occurs somewhat later than the core self actually initiating it. Such confusion is avoided if we use different terms to refer to the different levels of self-experience. We might, for example, call the re-represented (prefrontal) self of everyday cognition the “declarative” noetic self, and the primary affective (brainstem) state of being might be called the “core” anoetic affective self. The intermediate (posterior cortical) somatosensory-motor self might then be called the “bodily” self. With autonoetic consciousness we can have vast varieties of idiographic selves [49]. 

Our major conclusion may now be stated thus: the core self, synonymous with Freud’s “id”, is the font of all consciousness; the declarative self, synonymous with Freud’s “ego”, is unconscious in itself. However, because the ego stabilizes the core consciousness generated by the id, by transforming affects into object representations, and more particularly verbal object re-representations, we ordinarily think of ourselves as being conscious in the latter sense. This obscures the fact that our conscious thinking (and exteroceptive perceiving, which thinking re-represents) is constantly accompanied by low level affects (some kind of residual “free energy” from which cognitive consciousness was constructed during developmental psychogenesis). However, the underlying primary, affective form of consciousness is literally invisible, so we have to translate it into perceptual-verbal imagery before we can “declare” its existence. 

The dumb id, in short, knows more than it can admit. Small wonder, therefore, that it is so regularly overlooked in contemporary cognitive science. But the id, unlike the ego, is only dumb in the glossopharyngeal sense. It constitutes the primary stuff from which minds are made; and cognitive science ignores it at its peril. We may safely say, without fear of contradiction, that were it not for the constant presence of affective feeling, conscious perceiving and thinking would either not exist or would gradually decay. This is just as well, because a mind unmotivated (and unguided) by feelings would be a hapless zombie, incapable of managing the basic tasks of life.

## 11. If the Id Is Conscious…

The realization that Freud’s id is intrinsically conscious has massive implications for psychoanalysis, biological psychiatry, and our understanding of the nature of mind. This turn of events could be profound, not least because when Freud famously proclaimed “where id was, there shall ego be” ([57], p. 80) as the therapeutic goal of his “talking cure”, he assumed that the ego enlightened the id. It now appears more likely that the opposite happens; reflexive “talking” is apt to dampen and constrain core consciousness. How is this fact to be reconciled with the stated aim of psychoanalytic therapy, namely the undoing of repressions? And what are the implications for other approaches to psychotherapy and psychiatry? 

To just begin to answer this question, it seems reasonable to suggest that repression must involve withdrawal of declarative awareness (*autonoetic* experience) from cognition. This has the effect of reducing an “episodic” cognitive process to an “associative” one. The subject of repression still activates the representations in question (the repressed “object relationships”), but the associative links no longer attract reflexive awareness. We recall that this is the original purpose of ego development: the goal of all learning is automatized mental processes, increased predictability, and reduced uncertainty or “surprise”. It is the biological salience of prediction errors—probably mentally mediated by a variety of feelings in addition to actual surprise—that requires the affective presence of the id (of the biological self). As soon as the ego has mastered a mental task, the relevant associative algorithm is automatized. This, then, could be the mechanism of repression: it consists in a premature withdrawal of reflexive awareness, automatization of a mental-behavioral algorithm before it actually fits the bill. This would result in prediction error, and therefore the ongoing risk of the repressed material reawakening affective salience. This lays the foundations for a “return of the repressed”, the classical mechanism of neurosis. 

The therapeutic task of psychoanalysis, then, would be to undo repressions (to allow the affective distress associated with the repressed situation to emerge), in order to enable the reflexive subject to properly master it, and generate episodic representations adequate to the task, so that it may then be legitimately automatized.

Psychosis, on this model, entails an almost opposite mechanism. Psychotic states appear to flow from protracted failure to automatize predictive models of the world, again presumably due to their original inadequacy for the task. This does not account for the whole mechanism of psychotic states, but positive psychotic symptoms at least (the omnipotent “attempts at self-cure” as Freud called them) entail excessive salience [58]. These patients live in a perpetual state of surprise, against which they defend themselves with delusional certainties [59]. As with neurotic patients, the therapeutic task is therefore to help these patients develop more adequate solutions to the tasks of life, but in the case of psychosis we are confronted by a mind that actually knows “less than it feels it does”, rather than knowing “more than it admits”. The problem is the constant presence of the id—the constant requirement for the biological self to affectively evaluate the meaning of experience. The therapeutic aim “where id was there shall ego be” therefore seems more appropriate for psychosis than neurosis.

Since most readers of this journal are not psychoanalysts though, we will briefly apply our therapeutic suggestions to the current state of cognitive neuroscience. Considering its breathtaking neglect of the affective dimension of the mind, and thereby of core consciousness itself, the most reassuring observation we can make about contemporary cognitive neuroscience is that it is not suffering from a psychosis. Cognitive neuroscience is neurotic; it suffers from repressions rather than delusions; it “knows more than it admits” rather than “less than it feels”. Many aspects of mind, such as affective states, have been prematurely placed into the unconscious, even though they are experienced, but rarely acknowledged or measured (for a full discussion of issues, see [29] and accompanying commentaries). Our task is therefore to gently draw the attention of our cognitive colleagues to the constant prediction errors this gives rise to, and help them to tolerate the ensuing distress, so that a more realistic, affectively based, predictive model of the brain may emerge.

## 12. The Deepest Insight

The irony of the foregoing historical-scientific review is that Freud might appear to have done a disservice to psychology by denying consciousness its pride of place. But this is not really the case. 

Firstly, when Freud was confronted in 1938 by the behaviorist juggernaut that was about to sweep aside his life’s work, he remarked that consciousness was:

“…a fact without parallel, which defies all explanation or description. Nevertheless, if anyone speaks of consciousness we know immediately and from our most personal experience what is meant by it. One extreme line of thought, exemplified in the American doctrine of behaviorism, thinks it possible to construct a psychology which disregards this fundamental fact!”.

([26], p. 157)

In this spirit, we redirect attention here to the primacy of consciousness in mental life and brain research. This in no way diminishes the importance of deeply unconscious learning processes of the brain, *etc.* and the endless peppercorns of facts that have been generated by the “neuroscience revolution”; but it questions the attractions of ruthless reductionism, currently so popular in biological psychiatry, where investigators try to jump from brain facts to the cacophony of psychopathologies codified in successive DSMs, without explicit interest in the intervening role of the experienced mind itself. Future progress in this field requires us to recognize the fundamentally affective infrastructure of the mind, and its pivotal role in psychiatric disorders. We should be devoting at least as much effort to clarifying the neurodynamics of the primary affective processes in psychiatric disorders as we do to their mindless and seemingly endless neural and genetic correlates [60]. 

Secondly, we must recall that embedded within the many statements that Freud made to the effect that consciousness (by which he seemed mainly to mean declarative consciousness) was a cortical function, he always acknowledged the exceptional role of affect. For example:

“The question, ‘How does a thing become conscious?’ would be more advantageously stated: ‘How does a thing become preconscious?’ And the answer would be: ‘Through becoming connected with the word-presentations corresponding to it.’ These word-presentations are residues of memories; they were at one time perceptions, and like all mnemic residues they can become perceptions again. Before we concern ourselves further with their nature, it dawns upon us like a new discovery that only something that has once been a Cs. perception can become conscious, and that anything arising from within (apart from feelings) that seeks to become conscious must try to transform itself into external perceptions: this becomes possible by means of memory-traces”.

([17], p. 20, emphasis added)

In other words, although Freud thought that affects, too, were (interoceptive) cortical perceptions, he recognized that they were felt *directly*. He did not share the view that they first needed to be exteroceptively represented, or read-out, or “labeled” in working memory, to exist (which seems nowadays to be the most common viewpoint in the cognitive science of emotions). In fact, for Freud affects could not be represented in the same way that external objects were. This set them apart from all cognitive processes:

“It is surely of the essence of an emotion that we should be aware of it, *i.e.*, that it should become known to consciousness. Thus the possibility of the attribute of unconsciousness would be completely excluded as far as emotions, feelings and affects are concerned”.

([5], p. 177)

It is to be hoped that the neuroscientific facts reviewed here will help to make sense of this observation which, to Freud’s credit, he always acknowledged, notwithstanding the theoretical difficulties it must have caused him.

We will close with the observation that more essential to Freud’s successive models of the mind than the locus and extent of consciousness, was his fundamentally dynamic conception of it, coupled with the dimension of depth (or hierarchy) in the mind. This is why Freud repeatedly stated that the assumption of “two different states of cathectic energy in mental life: one in which the energy is tonically ‘bound’ and the other in which it is freely mobile and presses towards discharge” was the deepest insight he had ever gained:

“In my opinion this distinction represents the deepest insight we have gained up to the present into the nature of nervous energy, and I do not see how we can avoid making it”.

([5], p. 188)

This dynamic distinction is not only preserved in our affective neuroscience update of Freud’s views—along with much else—it is actually enhanced. The link between affectivity on the one hand and Helmholtz’s troublesome “free” energy on the other seems to provide a red thread through Freud’s work, linking him backwards to Helmholtz and forwards (via Feynman) to Friston. Considering this and the many other vistas opening up with the rediscovery of the embodied, instinctual brain—which must of necessity be constrained by the cognitive brain and its predictive modeling—it is difficult to imagine how the neuroscience and psychology of the future can be anything but neuropsychodynamic. As the cognitive science of the late twentieth century is complemented by the affective neuroscience of the present, we are breaking through to a truly mental neuroscience, and finally understanding that the brain is not merely an information-processing device but also a sentient, intentional being. Our animal behaviors are not “just” behaviors; in their primal affective forms they embody ancient mental processes that we share, at the very least, with all other mammals.

## 13. In Conclusion

Our goal here was to re-establish a primary-process affective foundation of mind to the higher mental apparatus that is receiving the lion’s share of attention in cognitive science and cognitive neuroscience, as well as consciousness and psychoanalytic studies. We chose to frame our argument in classical Freudian psychoanalytic theory, since during the 20th century, perhaps he came closest to the vision we have shared. Although he, in the modern vein, situated consciousness on top of the brain, the weight of evidence now indicates that raw affects arise from the “basement” of the brain. Our argument has been largely restricted to the basement, and we recognize that when it is interfaced with unconscious secondary learning-memory and the resulting affectively energized cognitive thought processes, there will be vast additional complexities to be faced, along with the possibility of many mereological fallacies (part-whole confusions and conflations), as discussed superbly by Bennett and Hacker, in their exceptional 2003 book [61].

Of course, primal emotions unfold in relation to individual lives, but they are built into the brain as nomothetic endogenous behavioral and affective resources of the organisms. We have not sought to address how these ancestral powers of the mind percolate through the subsequent idiographic layers of brain-mind emergence. Freud attempted to do that, but future cognitive neuroscience and related neurophenomenological consciousness studies, will need to flesh out those processes, with a full recognition that the bottom-up developmental-epigenetic emergence of minds needs to provide a solid foundation for the vast complexities of the automatized top-down regulations and effortful conscious mental controls that a mature (fully-constructed) mental apparatus permits and promotes (Figure 1). 
